# Next-generation Candida albicans recombinant Als3p and Hyr1p dual antigen vaccine for invasive Candida infections

**DOI:** 10.21203/rs.3.rs-7270678/v1

**Published:** 2025-09-05

**Authors:** Shakti Singh, Eman G. Youssef, Ashley Barbarino, Haley Hautau, Sunna Nabeela, Teclegiorgis Gebremariam, Sondus Alkhazraji, Gary Ostroff, Dennis Christensen, Terrence Cochrane, Ashraf S. Ibrahim

**Affiliations:** The Lundquist Institute at Harbor-UCLA Medical Center; The Lundquist Institute at Harbor-UCLA Medical Center; The Lundquist Institute at Harbor-UCLA Medical Center; The Lundquist Institute at Harbor-UCLA Medical Center; The Lundquist Institute at Harbor-UCLA Medical Center; The Lundquist Institute at Harbor-UCLA Medical Center; The Lundquist Institute at Harbor-UCLA Medical Center; University of Massachusetts Chan Medical School; Croda Inc; Vitalex Biosciences LLC; The Lundquist Institute at Harbor-UCLA Medical Center

## Abstract

*Candida* species, including *Candida albicans* and *Candida auris*, represent a growing public health concern due to their increasing prevalence and resistance to antifungal agents. *C. albicans* is known for causing both superficial and invasive infections, while *C. auris* is a newly emerged, multidrug-resistant pathogen responsible for severe hospital outbreaks with a high mortality rate of ~ 60% in bloodstream infections. Vaccine candidates targeting *C. albicans* hyphal cell wall proteins Als3p and Hyr1p have shown protective efficacy in mice. NDV-3A, an alum-formulated Als3p-based vaccine, protects against recurrent vulvovaginal candidiasis in women. We earlier showed that both Als3p and Hyr1p have orthologs in *C. auris*, and that the NDV-3A vaccine, alongside an anti-Hyr1p monoclonal antibody, protect mice from lethal *C. auris* candidemia. Here, we optimized Als3p and Hyr1p dual antigen vaccine formulations with the clinical-stage adjuvant CAF01, demonstrating robust immunity and CD4 T celldependent protection against lethal *C. albicans* and *C. auris*. The vaccine formulations also showed enhanced protective efficacy when combined with antifungal drugs. This study highlights the potential of the CAF01-formulated Als3p/Hyr1p dual antigen vaccine in providing durable protective immunity against systemic and mucosal *C. albicans* and cross-protection against systemic multidrug-resistant *C. auris* infections.

## INTRODUCTION

*Candida* is the most common cause of invasive fungal infections in countries with advanced medical technologies [1]. In particular, invasive *Candida* infections are predominant in the immunocompromised patient population admitted to intensive care units (ICU) and have invasive medical devices (e.g. catheters, ventilators, and breathing tubes) [2–34]. *Candida* spp. (including those caused by the predominant *C. albicans*) are now statistically tied with *Enterococcus* as the third most frequent nosocomial bloodstream isolates [35–37], surpassing the incidence of bacteremia caused by *Escherichia coli* or *Klebsiella* species. Even with antifungal therapy, disseminated candidiasis has a ~ 40% mortality rate [38, 39]. The cost associated with hematogenously disseminated candidiasis is estimated to be $2–4 billion/year in the United States [40–42]. *Candida auris* has emerged as a significant threat to global health, having been reported in > 140 countries. *C. auris* clinical isolates are highly drug-resistant, which makes them challenging to eradicate once an infection has been established leading to increased healthcare costs [43] and morbidity and associated mortality of ~ 60% [44].

The past few decades provide evidence that the development of resistance to antibiotics is inevitable. Thus, it is essential to develop novel anti-infective approaches that do not rely solely on a drug’s antibacterial action. In this context, boosting the patient’s immunity through vaccines and immunomodulators is a promising new adjunctive and/or alternative therapeutic concept. Vaccines are the most effective and practical strategy for eliminating certain diseases, as exemplified by smallpox[45].

We have characterized *C. albicans* Agglutinin-like sequence-3 protein (Als3p, an adhesin and invasin factor for host tissues)[46–48] and Hyphal-regulated protein (Hyr1p, a neutrophil evading factor) [49, 50]. Our recombinant Als3p-based alum-adjuvanted (NDV-3A) vaccine[50] and a recombinant Hyr1p-based vaccine[49] elicited robust T- and B-cell responses and protected against murine *C. albicans* hematogenously disseminated candidiasis (including non-albicans spp.) and vulvovaginal candidiasis. In a Phase 1b/2a trial, a single dose of the recombinant Als3p antigen adjuvanted in Alhydrogel^®^ (NDV-3A) was found to be safe, immunogenic and protected women < 40 years of age from recurrent vulvovaginal candidiasis (RVVC) up to 12 months of follow up. However, this protection was modest with 42% of the vaccinated versus 22% of the placebo patients were symptom-free[51].

We recently identified three Als3 orthologs on the *C. auris* cell wall that share remarkable structural and functional similarities with *C. albicans* Als3p. NDV-3A vaccination significantly protected mice from a lethal hematogenously disseminated *C. auris* infection [52]. *C. auris* also has 8 orthologs of Hyr1p, of which two proteins are present in all four clades of *C. auris*. These Hyr1-orthologs have high predicted structural similarity with Hyr1p and contain a central adhesive domain, N-terminal substrate-binding domain, and GPI-anchor (like Hyr1p) [53]. Anti-Hyr1p monoclonal antibodies raised by us [54] and others [55], bind to *C. auris* surface and protect mice from *C. auris* disseminated infection. Furthermore, we first reported that Als3p and Hyr1p specific antibodies cross-react and bind to *C. auris* and prevent its adhesion to the plastic and biofilm formation potentially by blocking these adhesins. These findings were confirmed by subsequent studies emphasizing the role of Als and Hyr1 protein orthologs in *C. auris* adhesion, aggregation, biofilm formation, and skin colonization [56–59].

Our next-generation fungal vaccine development efforts leverage newer adjuvant systems and combine *C. albicans* recombinant Als3p and Hyr1p antigens to maximize immunogenicity and protection in preclinical models of infection. The primary goal of this endeavor was to achieve a balanced, broader, and robust antibody and T-cell immune response against each vaccine antigen. We generated over 100 vaccine formulations utilizing different ratios of Als3p and Hyr1p antigens mixing with a variety of adjuvants shown to be safe in preclinical studies, clinical trials or approved by regulatory agencies for vaccine development. These adjuvants included alum (Adjuphos^™^, comparator), Cation Adjuvant formulation-01 (CAF01^™^, Serum Staten Institute, Denmark) [60, 61], BDX100 and BDX300 (Inspirevax, Kirkland, QC, Canada) [62], Glucan Chitosan particles (GCP, developed by University of Massachusetts, MA, USA) [63], and MF59 (developed by Novartis) [64, 65]. We embarked on the immunogenicity evaluation of these dual antigen vaccine formulations in outbred CD-1 mice and compared the antigen-specific antibody and T cell immune responses among different adjuvants. Based on immunogenicity screening, several formulations were advanced for protective efficacy testing against invasive *Candida spp*. infections and vulvovaginal candidiasis by *C. albicans* in clinically relevant animal models. Our study is the first to report the comparative immunogenicity of various approved, pre-clinical, and clinical-stage adjuvant formulations and evaluation of *C. albicans* Als3p and Hyr1p dual antigen vaccine against candidiasis due to *C. albicans* and MDR *C. auris* in mice.

## RESULTS

### Manufacturing C. albicans Recombinant Als3p and Hyr1p Vaccine Antigens

**Figure 1:**
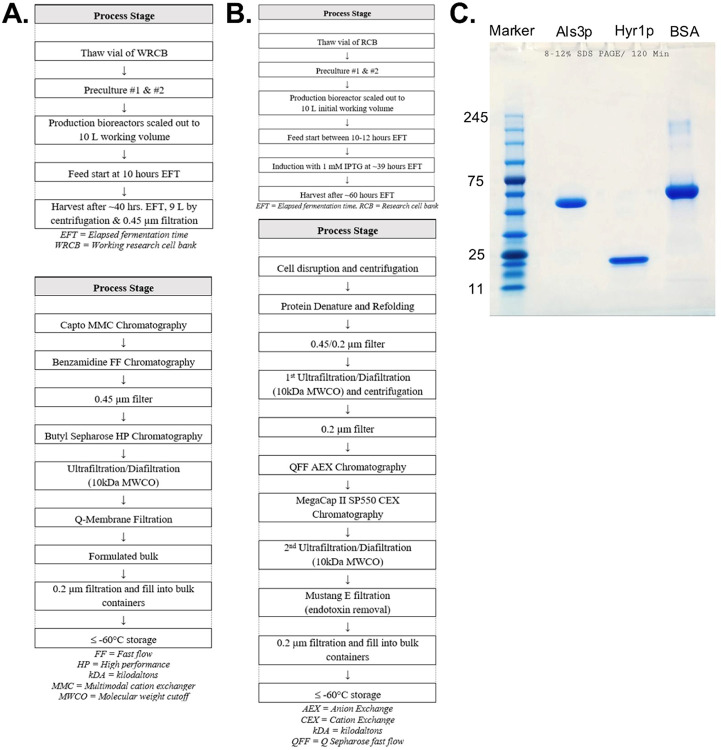
The manufacturing processes of *C. albicans* Als3 and Hyr1 antigens. The flowchart of the Upstream (Upper) and Downstream (Right) manufacturing process of recombinant Als3p (**A)** and Hyr1p **(B)**protein expressed and purified from *S. cerevisiae* and *E.coli*, respectively, is outlined in the flow diagram. **(C)**. SDS-PAGE analysis of recombinant Als3p and Hyr1p antigens. Single clean protein bands of each protein indicate high purity and integrity of the antigens.

The recombinant N-terminal regions of Als3p (18–450 amino acids) and Hyr1p (154–350 amino acids) were expressed in *Saccharomyces cerevisiae* FY03–1 and *Escherichia coli* BL21, respectively. For Als3p manufacturing, we used a previously developed Research Cell Bank (RCB) to establish a Working Research Cell Bank (WRCB) and conducted media optimization processes to enhance the growth and stability of the strain (Fig. 1A). For Hyr1p manufacturing, a vial of RCB stock was expanded into a 10-liter fermentation to determine the suitability of the selected clone for producing Hyr1p and plasmid stability at the end of fermentation (Fig. 1B). Finally, Als3p and Hyr1p were manufactured under non-Good Manufacturing Practice (GMP) conditions in a 10L bioreactor scale with upstream (USP) and downstream processes (DSP) applicable for *S. cerevisiae* and *E. coli* cell lines, respectively. The manufacturing and protein purification processes were monitored via preliminary in-process controls and tests shown in Fig. 1 and **Table S1**. The purity and integrity of both Als3p and Hyr1p were verified by SDS-PAGE analysis (Fig. 1C). The purified antigens produced in Good Laboratory Practice (GLP)-compliant conditions were filled in vials and stored at −80°C.

### Dual antigen vaccine immunogenicity is dependent on adjuvant selection

**Figure 2:**
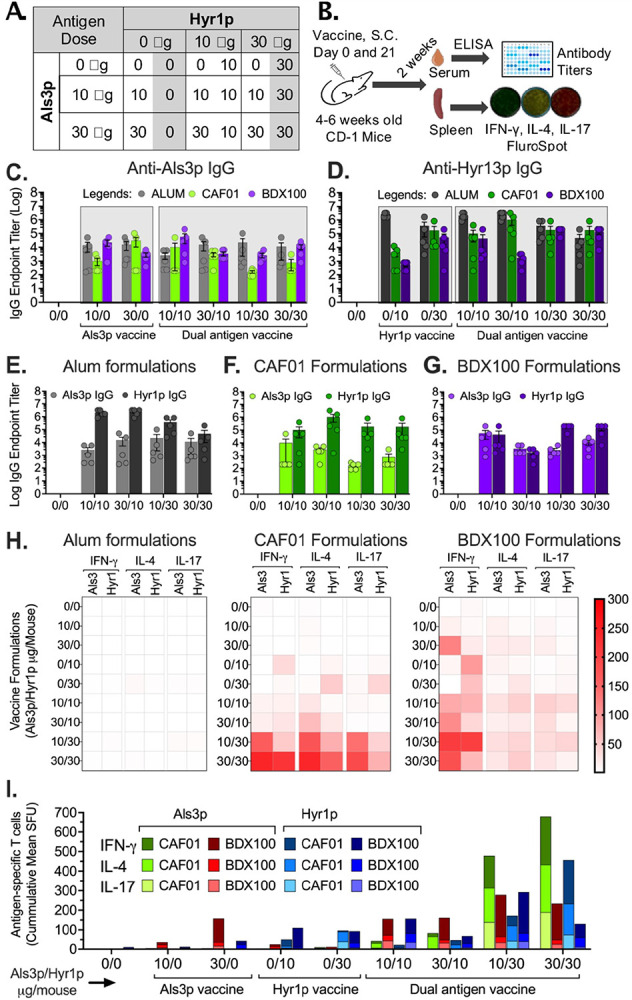
Immunogenicity of dual antigen vaccine formulated with Alum, CAF01, or BDX100 adjuvants. **(A)**. Combination of Als3p/Hyr1p doses by checkerboard method. **(B)**. Schematic of the experimental design for determining vaccine-induced immunogenicity is shown. The ICR CD-1 mice (N= 5/group) vaccinated SC (Alum, CAF01) or IN (BDX100) with different vaccine formulations (Als3 and Hyr1 antigens ratio on the x-axis) on days 0 and 21. Two weeks after the final vaccination, serum antigen-specific IgG titers and T cells were evaluated using ELISA and FluroSpot assay, respectively. Comparison of **(C)**. Anti-Als3 and **(D)**. anti-Hyr1 IgG endpoint titers in Alum, CAF01 or BDX100 adjuvant-antigen formulations. Anti-Als3 and anti-Hyr1 IgG endpoint titers in **(E)**. Alum, **(F)**. CAF01, **(G)**. BDX100 adjuvant-antigen formulations. **(H)**. Heat map showing the mean frequency (n=5 mice/cell/formulation) of Als3 or Hyr1-specific Th1, Th2, and Th17 cells (IFN-gamma, IL-4 or IL17 producing cells) in mice vaccinated with Alum, CAF01 or BDX100 vaccine formulations. Each row represents data from each vaccine formulation. **(I)**. Bar graph showing Als3 or Hyr1-specific Th1, Th2, and Th17 cells.

To optimize a dual antigen vaccine, we tested several Als3p/Hyr1p antigen ratios coupled with different adjuvants for their immunogenicity in mice. We used alum, CAF01, BDX100, BDX300, GCP or MF59 adjuvants to formulate the vaccine because of their prior safety profiles in vaccine development. Alum formulations were used as a comparator since the previous generation vaccine, NDV-3A, was formulated with Als3p adjuvanted with alum. Encapsulation of Als3p and Hyr1p antigen in GCP was confirmed by SDS-PAGE analysis (**Figure S1**). We tested 0, 10, or 30 μg of Als3p/dose with 0, 10, or 30 μg of Hyr1p/dose, yielding 9 antigen ratios by the checkerboard method for each adjuvant (Fig. 2A). BDX100 and BDX300 formulations were administered intranasally, and all other adjuvant formulations were administered subcutaneously on days 0 and 21. We evaluated the antigen-specific antibody and T-cell responses two weeks after the final immunization on day 35 (Fig. 2B). The anti-Als3p and Hyr1p IgG titers of the dual antigen vaccine formulations using alum, CAF01, or BDX100 are shown in Fig. 2C,D. The immunogenicity data for all other formulations is available in Supplementary **Figure S2–S3**.

Anti-antigen IgG titers depended on the adjuvant used and not on the antigen ratio used in the formulations, with adjuvant inducing a robust antibody titers. For example, GCP vaccine formulations induced the highest anti-Als3p IgG antibody titers, followed by MF59, Alum, CAF01, BDX100, and BDX300 (Fig. 2C, S2A). Anti-Hyr1p IgG titers were the highest for the alum formulations, followed by CAF01, BDX100, BDX300, and MF59 adjuvant formulations (Fig. 2D, S2B). Relative antigen dosage in the vaccine formulations did not influence the anit-Als3p or anti-Hyr1p-specific antibody titers in any adjuvant except the CAF01 adjuvant, which showed reduced anti-Als3p IgG titters with higher Hyr1p dose of 30 μg in the vaccine formulation (Fig. 2E–G, S2C). Furthermore, the dual vaccine formulations induced similar or higher anti-Als3p or Hyr1p IgG titers than the mono-antigen vaccine formulations, and this effect was not altered by the antigen dosage in the vaccine formulation. Collectively, these results show that the anti-Als3p- or anti-Hyr1p-specific IgG titers were not negatively influenced by increasing the relative Hyr1 or Als3p antigen dose in the vaccine formulation. Therefore, the Als3p and Hyr1p are not antagonistic to each other in the dual antigen vaccine formulations (Fig. 2C–D).

Alum coupled with Hyr1p mono-antigen formulation at 10 μg/dose induced higher anti-Hyr1p IgG titers compared to comparative dose mixed with either CAF01 or BDX100 adjuvants. Moreover, CAF01 and BDX100 adjuvant formulations showed an antigen dose-dependent increase in anti-Hyr1p IgG titers, which became similar to alum mono-Hyr1 antigen formulations at 30 μg/dose, indicating more antigen dose dependency with these adjuvants (Fig. 2D). In general, anti-Hyr1p IgG titers were higher than anti-Als3p IgG titers when alum, MF59, BDX100 or BDX300 were used as adjuvants, and this trend did not change with increasing Als3p antigen relative to Hyr1p antigen dosage (Fig. 2E–G, S3A-B). In contrast, when GCP was used as an adjuvant, anti-Als3p IgG titers where noticeably higher than anti-Hyr1p IgG at all Als3p/Hyr1p antigen ratios (**Figure S2C**).

We also examined the T cell immune responses in the splenocytes using a triple color IFN-g (Th1), IL4 (Th2), and IL17(Th17) FluroSpot assay. Alum-based vaccine formulations failed to elicit CD4 + T cell responses, while all other vaccine formulations induced detectable Th1, Th2, and Th17 immune responses. Among these, CAF01 formulations generated robust and balanced Th1, Th2, and Th17 immune responses in a vaccine antigen dosage-dependent manner, targeting both Als3p and Hyr1p antigens. BDX100 formulations predominantly elicited Th1-biased responses that equally targeted Als3p and Hyr1p antigens, whereas BDX300 induced balanced Th1, Th2, and Th17 responses that were proportionally equivalent but of lower magnitude compared to CAF01. A notable difference between BDX100 and BDX300 was the stronger Th1 response observed with BDX100. GCP formulations elicited strong Th1- and Th17-skewed immune responses, favoring the Als3p antigen. In contrast, MF59-based formulations generated weak Th1, Th2, and Th17 responses regardless of the Als3p and Hyr1p antigen dosage. Overall, CAF01, BDX100, and BDX300 formulations produced proportionally similar Th1, Th2, and Th17 responses specific to Als3p and Hyr1p, with the magnitude of responses generally dependent on antigen dosage (Fig. 2H–I, S3C).

### The dual antigen CAF01 vaccine is highly efficacious against invasive candidiasis

**Figure 3:**
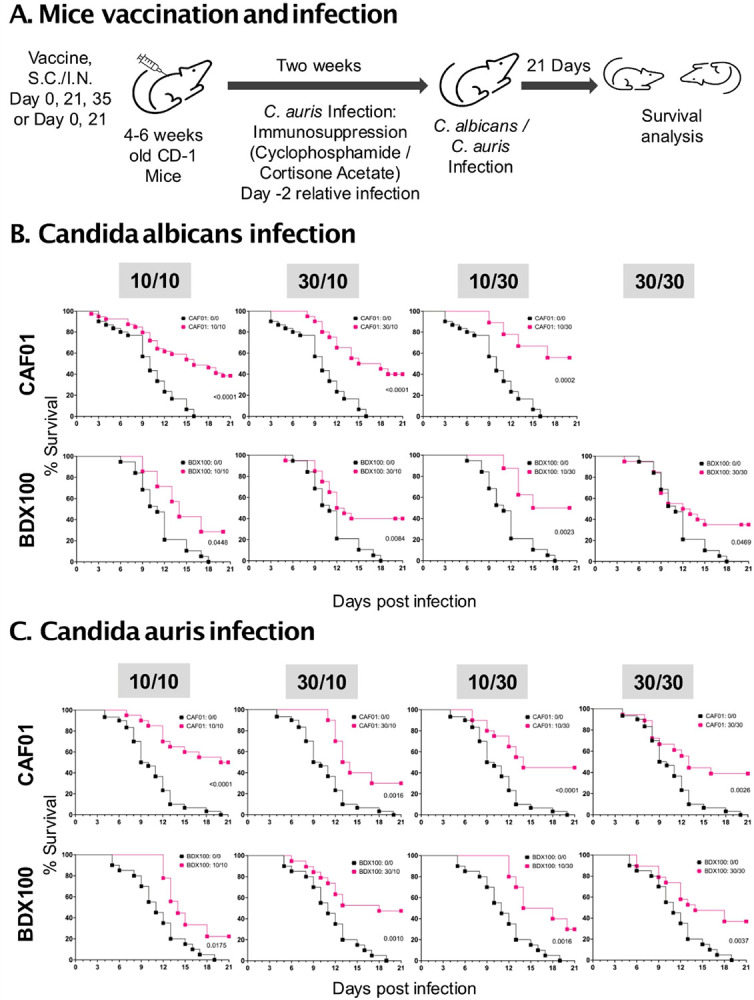
Survival efficacy of the Als3p/Hyr1p dual antigen vaccine against invasive candidiasis due to *C. albicans* and *C. auris*. **A)**. Experimental design for the *in vivo* survival efficacy. Mice were vaccinated two or three times with Als3p/Hyr1p dual antigens formulated with CAF01 or BDX100 adjuvant. Two weeks after the final vaccination, mice were infected intravenously with 2X10^5^ cells/mouse of *C. albicans* or 5X10^7^ cells/mouse of *C. auris*. For *C. auris* infection, the mice were immunosuppressed on day −2 relative to infection. **(B)**. Vaccine-induced survival efficacy against *C. albicans* infection after two booster immunizations. **(C)**. Vaccine-induced survival efficacy against *C. auris* infection after one booster immunization. Data are pooled from 2–5 repeated experiments with N=10 mice/group/experiment. Mice survivals were compared by the Mantel-Cox test, and p<0.5 was considered statistically significant.

We vaccinated mice with dual Als3p/Hyr1p antigens at 10μg/30μg or 30μg/30μg ratio formulated with alum, CAF01, GCP, or MF59 adjuvant on days 0 and 21 through intramuscular. The same antigen ratios were used, mixed with BDX100 and administered intranasally on days 0 and 21. Two weeks after the final vaccination, mice were intravenously infected with *C. albicans* or *C. auris*. For *C. auris* infection, mice were immunosuppressed at day − 2 relative to the infection. Infected mice were monitored for protective efficacy by survival at day 21 as the primary endpoint (Fig. 3A).

Among all the adjuvant formulations tested, only CAF01 and BDX100 vaccine formulations showed significant protection against both hematogenously disseminated *C. albicans* and *C. auris* infections with 36 to 42% survival (12–14 days of median survival time [MST]) vs. the placebo group showing 0% survival (8–11 days of MST) (**Figure S4**).

Based on the immunogenicity profile and preliminary efficacy studies, we prioritized CAF01 and BDX100 dual antigen vaccine formulations to further optimize and evaluate their protective efficacy against *C. albicans* and *C. auris* infections. CAF01 or BDX100 vaccine formulations with Als3p/Hyr1p dual antigens at 10μg/10μg, 30μg/10μg, 10μg/30μg or 30μg/30μg, were administered on days 0 and 21 (two vaccinations or 1 booster) or days 0, 21, and 35 (three vaccinations or 2 booster) as described above (Fig. 3A).

#### Efficacy against lethal hematogenously disseminated C. albicans infection.

Two vaccinations (one booster) with CAF01 formulations showed 10–32% survival (9–12 days MST) vs. placebo with 0% survival efficacy (8 days MST) against *C. albicans* disseminated infection (p = < 0.0001 to 0.099). Using three doses of the CAF01 vaccine formulations (two boosters) further significantly enhanced survival efficacy to 40–56% overall survival by day 21 and prolonged MST to 17 - >21 days (Fig. 3B, S5A). For BDX100 formulations, two vaccinations with the dual antigens showed 10%−40% overall survival efficacy with 10 to 11 days MST vs. 0% survival and 8 days MST for placebo. Similarly, three immunizations further enhanced the survival efficacy demonstrated by different ratio of the dual antigen vaccine, showing 29–50% survival efficacy with 13 to 18 days MST (Fig. 3B, S5A).

#### Efficacy against lethal hematogenously disseminated C. auris infection.

We also evaluated the efficacy of CAF01 and BDX100 vaccine formulations using one or two booster vaccination experiments. CAF01 formulations (Als3p/Hyr1p: 30μg/30μg, 10μg/30μg, 30μg/10μg, and 10μg/10μg) with one booster showed 30–50% overall survival and 13 to 21 days of MST vs. 0% survival and 9 days MST for placebo mice (CAF01-vaccinated without antigens) (Fig. 3C, S6). Similarly, using the same ratios of antigens coupled with BDX100 as a one booster vaccine resulted in an overall survival of 22–47% with 14 to 18 days of MST vs. 0% survival and 10 days of MST for placebo (Fig. 3C, S6). Two booster vaccinations with CAF01 or BDX100 formulation did not further enhance the survival efficacy (10–40% with 11 to 14 days of MST) (Fig. 3C, S6).

### The dual antigen CAF01 vaccine prevented weight loss and tissue microbial burden

**Figure 4:**
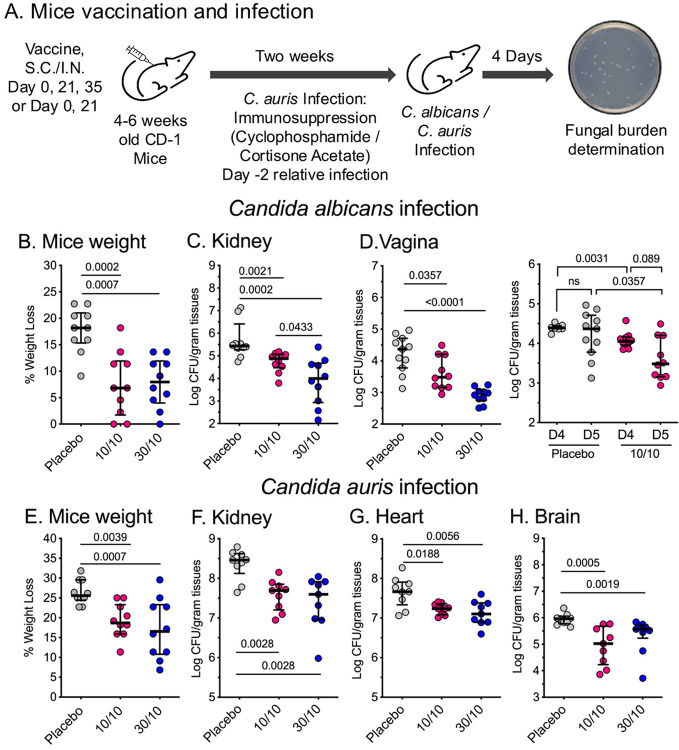
Tissue fungal burden in *C. albicans* and *C. auris* infected mice vaccinated with CAF01 Als3p/Hyr1p dual antigen vaccine. **(A)**. Experimental design for the tissue fungal studies is shown. ICR CD-1 mice were vaccinated two or three times with Als3p/Hyr1p dual antigens at 10μg/10μg or 30μg/10μg dose formulated with CAF01 adjuvant. Two weeks after the final vaccination, mice were infected intravenously with 2X10^5^ cells/mouse of *C. albicans* or 5X10^7^ cells/mouse of *C. auris*. For *C. auris* infection, the mice were immunosuppressed on day −2 relative to infection. Four days (hematogenously disseminated infections) or five days (VVC) post-infection, the mice were euthanized, and tissue fungal burden/gram tissues in target organs of vaccinated or placebo mice were determined. After 4 days of infection, the mice’s weight was also measured to assess their health status. **(B)**. Weight loss post-*C. albicans* infection, **(C)**. Kidney fungal burden in *C. albicans* infected mice, **(D)**. Vaginal *C. albicans* burden, **(E)**. Weight loss post-*C. auris* infection, **(F)**. Kidney, **(F)**. Heart, and **(F)**. Brain *C. auris* burden. Mice weights and tissue fungal burdens were compared between vaccinated and placebo mice (N=9–10/group) by the Mann-Whitney Test (Median + IQR).

These studies showed that CAF01 formulations, specifically Als3p/Hyr1p: 30μg/10μg, and 10μg/10μg afforded superior protective efficacies to BDX100 formulations against *C. albicans* and *C. auris* disseminated infection after two boosters and one booster vaccination, respectively. Thus, we further tested the effect of vaccination with these CAF01 formulations on the fungal burden of target tissues. Mice were vaccinated and infected with *C. albicans* (after two boosters) or *C. auris* (after one booster) as described earlier and euthanized at day 4 post-infection to enumerate tissue fungal burden. Mice weight was also recorded as a measure of the overall progression of infection and health status (Fig. 4A).

For *C. albicans*, the fungal burden in the kidneys was determined, as this organ is the primary target organ [66]. In a subset study and since the NDV-3A was protective against murine VVC[66], vaccinated mice were infected intravaginally, and tissue fungal burden was determined in vaginal tissues on days 4 and 5 post-infection. For *C. auris*, the kidney, heart, and Brain were determined on day 4 post-infection.

Both CAF01 Als3p/Hyr1p 10μg/10μg and 30μg/10μg vaccine formulations prevented significant weight loss compared to the placebo group in *C. albicans* or *C. auris* infected mice (p < 0.004) (Fig. 4B, E). Further, CAF01 10μg/10μg and 30μg/10μg vaccine formulations significantly reduced tissue *C. albicans* burden in the kidney and vagina by 0.5–1.5 log compared to the placebo group (p < 0.05) with the formulation of 30/10 appearing more effective in protecting against *C. albicans* infections (Fig. 4C, D). In *C. auris* infection model, CAF01 vaccine formulations reduced fungal burden by 0.5–1.0 log compared to the placebo group (p < 0.02) (**Figure F-H**). Therefore, CAF01 Als3p/Hyr1p 10μg/10μg and 30μg/10μg formulations showed superior efficacies in both *C. albicans* and *C. auris* infection models and required two booster vaccinations for *C. albicans* protection, while a single booster vaccination was sufficient to afford the significant protective efficacy against *C. auris*.

### CD4 T cells and to a lessor extent antibodies are required for the Als3p/Hyr1p + CAF01 vaccine protection

We investigated the role of humoral and adaptive immunity in the Als3p/Hyr1p + CAF01 vaccine-mediated protection using the 10μg/10μg vaccine formulation. Specifically, we conducted passive serum transfer and CD4 T cell depletion experiments. For *C. auris*, the mice were immunosuppressed, as described earlier, while immunocompetent mice were used for *C. albicans* model. For the passive serum transfer experiment, sera from 3 times (for *C. albicans*) or 2 times (for *C. auris*) vaccinated mice with Als3p/Hyr1p + CAF01 vaccine or those vaccinated with CAF01 alone were administered intraperitoneally to naïve infected mice at 2 and 168 hrs days post-infections and survival of mice was monitored for 21 days. For the CD4 T cell depletion experiments, we vaccinated mice with Als3p/Hyr1p + CAF01 (10μg/10μg) as above and then used anti-CD4 antibodies to deplete mice from CD4 T cells prior to infecting them with either *C. albicans* or *C. auris*. Survival of mice was documented over a 21-day period. CD4 T cell depletion was confirmed by determining the number of CD4 T cell population in both spleen and lymph nodes of representative mice treated with anti-CD4 IgG-treated mice vs. or isotype-matched control IgG (**Figure S7**) [52].

**Figure 5:**
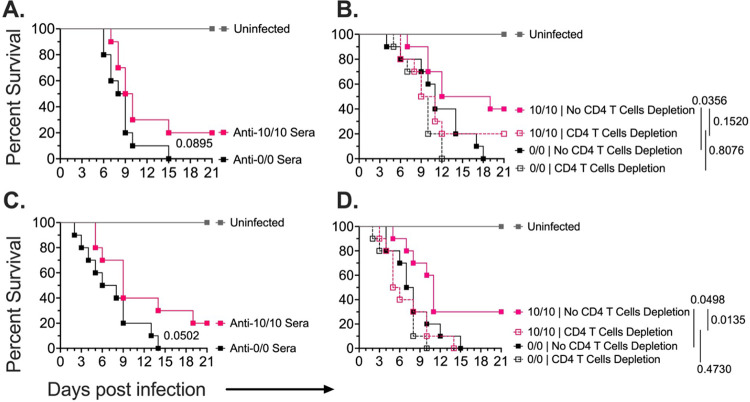
Mechanism of CAF01 dual antigen vaccine-mediated protection. Naïve mice or vaccinated mice (n= 10 mice/group) were infected with *C. albicans* or *C. auris*. For vaccination, the CAF01 dual antigen vaccine was administered on days 0, 21, and 35 (for *C. albicans* infection) or days 0 and 21 (for *C. auris* infection). Mice were immunosuppressed with cyclophosphamide and cortisone acetate for *C. auris* infection. Naïve mice (n= 10 mice /group) infected with **(A)**. *C. albicans* or **(C)**. *C. auris* received two intraperitoneal anti-sera injections at 2 and 168 hrs relative to infection. Vaccinated or placebo mice (n= 10 mice /group) received anti-CD4 or isotype control antibodies to deplete the CD T cells. The mice were infected with **(B)**. *C. albicans* or **(D)**. *C. auris*. Mice survivals were compared by Mantel-Cox test, and p<0.5 was considered statistically significant.

For *C. albicans* infection, adoptive transfer of sera from CAF01 10μg/10μg-vaccinated mice resulted in modest 20% survival with a MST of 9.5 days, compared to 0% survival and a MST of 8.5 days in placebo sera-transferred mice (p = 0.089) (Fig. 5A). In contrast, deletion of mice from CD4 T cell completely abolished protection afforded by the CAF01 10μg/10μg vaccine in non-depleted CD4 T cells (40% survival and an MST of 15.5 days versus 0% survival and an MST of 11 days in placebo mice [p = 0.035], while CAF01 10μg/10μg vaccinated and CD4 T cell depleted mice had 20% survival and MST of 10 days, versus 0% survival and 10 MST of days for unvaccinated and CD4 T cell depleted mice [p = 0.81]). Collectively, these results highlight the critical role of CD4 T cells in vaccine-mediated protection against *C. albicans* (Fig. 5B).

Similarly, for *C. auris* infection, adoptive transfer of CAF01 10μg/10μg sera conferred 20% survival with an MST of 9 days, compared to 0% survival and an MST of 7 days in placebo sera-treated mice (p = 0.05) (Fig. 5C). In CD4 T cell depletion experiments, the protection afforded by the CAF01 10μg/10μg vaccine in non-CD4 T cell depleted mice (30% survival and MST of 11 days for vaccinated mice vs. 0% survival and a MST of 7 days in placebo mice [p = 0.049]), was completely reversed in CD4 T cell depleted mice (0% survival and MST of 5 days of both vaccinated and placebo mice [p = 0.4546]) (Fig. 5D). These results confirm the critical role of CD4 T cells and point to a possible role for antibodies in the afforded protection elicited by the CAF01 10μg/10μg vaccine against murine *C. auris* candidemia.

### The dual antigen CAF01 vaccine induced long-lasting protective vaccine-induced immunity

To investigate the durability of the immunity afforded by the vaccine, the antigen-specific antibody and T-cell responses were tracked over 270 days following immunization with either 10μg/10μg vaccine formulation administered twice (days 0 and 21) or thrice (days 0, 21, and 35). After the final booster vaccination, mice were euthanized on days 14, 28, 90, 180, or 270 to evaluate antibody titers by ELISA and T-cell immune responses by FluroSpot assay.

**Figure 6:**
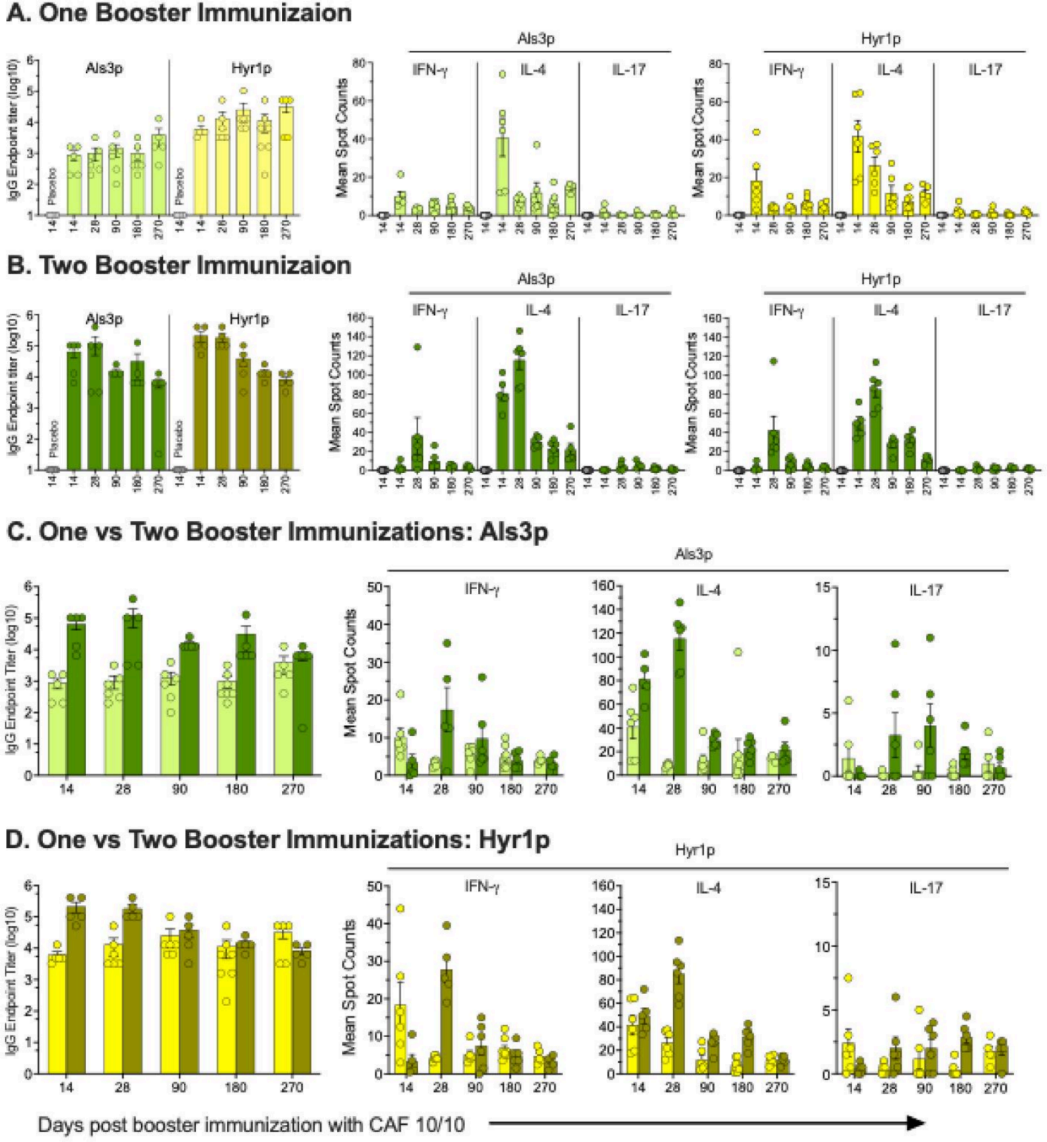
The durability of CAF01 dual antigen vaccine immunity. The ICR CD-1 mice were vaccinated subcutaneously with CAF01 dual Als3p/Hyr1p antigen at 10μg/10μg dose on **(A)**. days 0, 21, or **(B)**. days 0, 21, 35. The serum Als3p and Hyr1p-specific IgG endpoint titers and T cells were evaluated using ELISA and FluroSpot assay on days 14, 28, 90, 180, and 270 post-final vaccination. (C). Anti-Als3 and Hyr1p IgG endpoint titers and T-cell responses were compared between two and three vaccination schedules over the period of 270 days post-vaccination. Data presented as mean ±SE of N=5 mice/group.

Mice vaccinated with the 10μg/10μg formulation showed robust antibody responses against Als3 and Hyr1 antigens on day 14, which did not wane for up to 9 months of follow up. The two booster series induced ~ 1 log higher IgG antibody titer than the one booster series. For T-cell immunity, the one booster immunization resulted in Als3p and Hyr1p-specific T-cell responses that peaked at Day 14 and were similar in magnitude and biased towards Th1 and Th2. These T-cell specific responses declined after two weeks but were detectable for up to 9 months (Fig. 6A). For the two booster immunizations, Th1-antigen specific immune responses were similar to the one booster regimen (Fig. 6B), with two exceptions of responses being higher in magnitude and responses peaked at Day 28 instead of Day 14 for the one booster immunization (Fig. 6C, D). These results are concordant with data showing that mice intravenously challenged with *C. auris* 15 weeks following one booster regimen had a 30% 21-day survival vs. 0% for placebo and 14 days MST for vaccinated vs. 9 days for placebo (p = 0.0063) (**Figure S8**).

### Dual antigen CAF01 vaccine enhanced efficacy in combination with antimicrobial drugs

We also evaluated the efficacy of the CAF01 10μg/10μg vaccine formulation in combination with a subprotective dose of antifungal drugs currently used to treat *Candida* infections. Briefly, we vaccinated mice as above, and two weeks after the vaccination, mice were infected with the target *Candida spp*. Treatment with a suboptimal dose of the antifungal drug started on day + 1 post-infection. We found that drug treatment enhanced survival efficacies by 20–50% and prolonged MST by (> 4–16 days) compared to either drug or vaccine alone. However, the enhanced survival in the combination arm was not statistically different than the vaccine alone treatment (Table 1).

## DISCUSSION

*Candida* species, including *C. albicans* and *C. auris*, are becoming a significant public health concern due to their increasing prevalence and resistance to antifungal agents. *C. albicans* can cause both superficial mucosal and invasive infections. In contrast, *C. auris* has rapidly emerged as a multidrug-resistant pathogen, capable of causing severe hospital outbreaks with a high mortality rate of around 60% for bloodstream infections [67]. Its ability to persist in the environment and asymptomatically colonize patients’ skin further complicates control and prevention measures[68]. Factors such as immunosuppression, invasive medical procedures, and the emergence of antifungal resistance add to the complexity of managing *Candida* infections [69, 70]. Therefore, novel immunotherapeutics and vaccines are needed as viable adjunctive therapies to antifungal drugs.

Our previous efforts have focused on developing vaccine and immunotherapeutic antibody approaches targeting *C. albicans* adhesin/invasion protein Als3p and neutrophil evasion factor Hyr1p [71–73]. Alum-adjuvanted Als3p and Hyr1p recombinant protein-based vaccines have shown protection against invasive *C. albicans* infection in mice [71, 72]. NDV-3A, an alum-formulated Als3p-based vaccine, has been effective in protecting women from recurrent vulvovaginal candidiasis [51]. Additionally, the newly emerged multidrug-resistant *C. auris* harbors orthologs of both Als3p and Hyr1p. NDV-3A-induced cross-reactive immune responses and Hyr1p-epitope-based cross-reactive monoclonal antibodies have protected immunosuppressed mice against lethal *C. auris* infection [74, 75].

To further advance our efforts in developing the next generation of fungal vaccines, we explored combining both Als3p and Hyr1p in a dual antigen vaccine using more advanced and clinically safe adjuvant systems. This approach aims to induce broader and more durable and balanced antibody and T cell responses, represented by Th1, Th2, and Th17 cells targeting both Als3p and Hyr1p antigens. To achieve this, we standardized the manufacturing processes for recombinant Als3p and Hyr1p and verified the purity and integrity of each protein substance. To minimize the potential immunodominance of one antigen over the other, we used a checkerboard strategy to mix Als3p and Hyr1p protein antigens in different ratios. Combined with different adjuvant systems, these dual antigen ratios yielded dozens of dual antigen vaccine formulations. We screened these formulations *in vivo* for immunogenicity. Antigen-specific IgG endpoint titers and T cell responses were determined and compared within the formulation, among different antigen ratios for an adjuvant, and among different adjuvants.

The dual antigen vaccine formulations induced strong IgG titers, with GCP and MF59 adjuvant formulations performing particularly well. The relative antigen doses did not significantly influence the specific IgG titer for most adjuvant formulations, except for CAF01. This observation aligns with earlier research indicating that certain adjuvants may not exhibit antigen dose-dependent effects [76]. Further, the choice of adjuvant also significantly impacts the immune response. In our study, GCP formulations induced the highest anti-Als3p IgG titers, while alum formulations induced the highest anti-Hyr1p IgG titers. This finding is consistent with previous studies, which have shown that different adjuvants influence the magnitude and type of immune response [77, 78]. The dual antigen formulations induced similar or higher immune responses compared to single antigen formulations. This suggests that combining Als3p and Hyr1p does not negatively impact the immunogenicity of either antigen. This observation has significant implications for developing multivalent vaccines combining multiple antigens from similar or related pathogens to target multiple healthcare-associated pathogens. These results also highlight the critical role of adjuvants in vaccine formulation.

Long-lasting immunity is crucial for the effectiveness of vaccines, especially in preventing infections over extended periods. The sustained antibody titers and T-cell immune responses using CAF01 10μg/10μg vaccine over 9-months period suggest that the vaccine could provide long-term protection, reducing the need for frequent booster doses. Previously, we showed that a single dose NDV-3A vaccine induced robust and long-lasting IgG antibody titers in women, which significantly correlated with a lower incidence of recurrent VVC [79]. The three-dose series induced higher antigen-specific IgG antibody titers and Th1, Th2, and Th17 cells than the two-dose series. We observed a bias towards Th1 and Th2 responses after one booster immunization and a higher Th2 response with two booster immunizations. Interestingly, one booster showed peak antigen-specific Th1/Th2 T cell responses at day 14, compared to two booster vaccinations, which peaked at day 28. These results clearly show the critical role of booster doses and vaccination scheduling in the magnitude, quality, and kinetics of vaccine-induced immunity, aligning with previous studies in both mouse and human models [80–84].

We tested the protective efficacy of the top immunogenic dual antigen vaccine formulations and identified BDX100 and CAF01 as the most efficacious adjuvant formulations. Other adjuvant formulations, specifically GCP and MF59, failed to provide protection against both *C. albicans* and *C. auris* despite inducing robust antibody responses and with GCP also inducing a strong Th1/Th17 immune response. This suggests that a strong antibody response alone does not necessarily correlate with protective efficacy, a finding confirmed by our adoptive transfer experiments, which showed that antibodies are of limited value in conferring protection against hematogenously disseminated candidiasis caused by *C. albicans* or *C. auris*. Other immune mechanisms or factors must be involved in providing an effective defense against these fungal pathogens. One possibility is that the quality of the antibody response, rather than the quantity, is crucial for protection. The antibodies induced by these adjuvants may be of limited avidity, specificity or functionality. Additionally, the role of cellular immunity, particularly the contributions of CD4 T cell subsets, is more critical in combating these fungal infections, as previously reported by us and others [85, 86]. Our studies on CD4 T-cell depletion, which show the abrogation of the protective effect of the CAF01 Als3p/Hy1p vaccine in both models of *Candida* infection, confirm the importance of cell-mediated immunity.

We prioritized BDX100 and CAF01 vaccine formulations for further optimization and tested additional Als3p/Hyr1p antigen ratios with these two adjuvants. Our results indicate that both CAF01 and BDX100 vaccine formulations can provide significant protection against lethal *C. albicans* and *C. auris* infections. The efficacy of the vaccine formulations was dose-dependent, and lower antigen doses provided better protective efficacy against both fungal infections (e.g. doses of 10/10 μg or 10/30 μg of Als3p/Hyr1p had better mouse survival than 30/30 μg [Figure S5]). This could be potentially due to the induction of poor quality of antibody and CD4 T cells as previously reported [85, 86]. Further, three vaccinations with CAF01 formulations provided the highest survival rates and longest median survival times against *C. albicans* infection, suggesting that an additional booster can significantly enhance protective efficacy against *C. albicans* infection. For *C. auris* infection, one booster vaccination with either CAF01 or BDX100 formulations showed significant survival efficacy, and adding a second booster did not further improve survival rates, indicating a potential plateau in efficacy with additional doses. These differential reliance on number of vaccination could be attributed to the difference in the magnitude of long-lasting antibody titers and a balanced Th1/Th2 CD4 T cell response with one booster and Th2 biased CD4 cell responses in two booster immunizations. These results underscore the importance of optimizing both the antigen dose and vaccination schedule to achieve optimal protective outcomes. It is prudent to mention that if the vaccine is advanced into clinical testing, a single booster of the vaccine might be sufficient to elicit protection against *C. albicans* infections because the majority, if not all, humans are colonized with this yeast[87–89].

Based on the efficacy studies and the fact that CAF01 has been shown to be safe in several clinical trials[90–94]. We further prioritized CAF01-based dual antigen vaccine formulations and tested them in tissue fungal burden studies. The vaccination significantly reduced the tissue fungal burden in the kidney and vagina of *C. albicans* and, kidney, heart and brain of *C. auris* infected mice. These results also reflected the significantly less weight loss in vaccinated vs placebo control mice infected with *C. albicans* or *C. auris*.

The antibodies alone had a limited protective role compared to T cells in the vaccine-induced antifungal immunity. These results are aligned with our previous studies and emphasize the importance of cellular immunity, particularly the role of CD4 T cells in orchestrating and sustaining protective immune responses against both *C. albicans* and *C. auris* [75, 95].

It is imperative that any antifungal prophylactic or therapeutic approach be used in conjunction with clinically approved drugs. Thus, we investigated the potential of our optimized CAF01 10μg/10μg vaccine as an adjunct prophylactic approach. Our results showed that the antifungal drug combination treatment enhanced survival rates and median survival times compared to either the vaccine or the drug alone. These results suggest a potential synergistic effect between the vaccine and the antimicrobial drug, leading to improved outcomes in the infected mice. However, it is important to note that the study did not find a statistically significant difference between the vaccine alone and the combination treatment, possibly because of the limitation of the animal model used (e.g. more aggressive *Candida* infections in these experiments as highlighted by rapid early mortality post infection). Nonetheless, the enhanced survival observed with the combination treatment could be attributed to several mechanisms. The vaccine likely primes the immune system, providing an initial defense against the infection, while the antimicrobial drug helps to reduce the fungal burden, allowing the immune system to control the fungal pathogen more effectively. This combined approach may offer a more comprehensive strategy for combating *Candida* infections.

Future studies should focus on elucidating the specific and more in-depth immune mechanisms that confer protection against each fungal pathogen. Additionally, exploring the reasons behind the failure of GCP and MF59 to protect despite robust immune responses could provide valuable insights into the complexities of immune protection and guide the development of more effective vaccines. Further, understanding why certain adjuvants perform better with specific antigens can lead to the development of even more effective adjuvant-antigen combinations. Other studies should focus on optimizing the dosing and timing of both the vaccine and the antimicrobial drug to maximize their combined efficacy. Additionally, exploring the underlying immune mechanisms that contribute to the observed survival benefits could provide valuable insights into how to enhance the protective effects of the vaccine and drug combination. Finally, it is imperative to test the vaccine against *Candida* infections caused by other species, given their increasing number of clinical cases. These studies will help in the further optimization of the vaccine formulations with improved protective efficacy against lethal *Candida* infections, including MDR *C. auris*.

In conclusion, our data provide evidence that an improved second-generation dual antigen vaccine is more effective than the NDV-3A vaccine in providing a sustained immune response and long-lasting protection, which is likely to benefit patients at risk of hematogenously disseminated candidiasis. lasting protection.

## MATERIALS AND METHODS

### Antigens, Expression Systems, Cell banks, and Manufacturing

The Als3 antigen is a recombinant N-terminal region of an adhesin protein from *C. albicans* expressed in *Saccharomyces cerevisiae*. The expressed protein has 416 amino acids. Hyr1 constitutes a recombinant N-terminal portion (197 amino acids) of the native cell surface protein from *C. albicans*, that is expressed in an insoluble inclusion body form in *E. coli*.

For Als3p expression, a Master Cell Bank (MCB) of *S. cerevisiae* strain FY03–1 maintaining the vector pTEF1-S1Als3-2 (codon optimized for *S. cerevisiae*) was established earlier by Althea Technologies utilizing the parent strain DY150 (Clontech). Subsequently, this strain was further optimized to develop a Research Cell Bank (RCB) designated as Fy03–1/2um-full/TEF1p. This optimization yielded a more stable cell line due to the inclusion of the full 2μm origin sequence. A Working Research Cell Bank (WRCB) was prepared from the above RCB by Biodextris for the manufacturing process of Al3p. Briefly, one vial of Working Research Cell Bank containing *S.cerevisiae* FY03–1 producing Als3p was thawed and added to the primary shake flask, and then expanded into a secondary shake flask to further enlarge the working culture for bioreactor inoculation. Once the secondary shake flask reaches its set OD, the culture is used to inoculate the 10L production bioreactor, which is run for approximately 40 hours in fed-batch mode while the Als3 product is constitutively expressed. Once the harvest criterion is achieved, the biomass is separated from soluble expressed Als3 by centrifugation, with the product being clarified by depth filtration and 0.2μm filtration. Following harvest, the filtered concentrate is loaded onto a Capto MMC column for Als3 product capture, eluted, and flowed through a Benzamidine FF column for host cell protein reduction, followed by polishing on a Butyl HP chromatography step. The eluate from the Butyl HP step is then buffer exchanges and flowed through a Sartobind Q membrane adsorber and then is 0.2μM filtered, aliquoted, and stored at ≤ −60°C (Fig. 1).

For Hyr1p expression, an N-terminal region of the Hyr1 gene was bacterial codon-optimized, cloned in a bacterial proprietary plasmid expression vector (Nature Technologies Inc.), and transformed into a competent *E. coli* BL21 strain (New England Biolabs). Subsequently, the plasmid-transformed E. coli cell line underwent rounds of culture and clonal isolation to screen for promoter regulation, productivity, copy number, restriction map, and level of dimerization, resulting in the selection of a colony to establish the pre-RCB cell bank. Briefly, one vial of Research Cell Bank containing *E.coli* BL21 producing Hyr1 by promoter induction is expanded by shake flask to inoculate a 10L bioreactor in chemically defined media in the presence of Kanamycin. Once inoculated, the 10L production bioreactor is run in fed-batch mode for approximately 24 hours to increase biomass and then induced with IPTG for Hyr1 product expression over 24 hours. Once the harvest criterion is achieved, the biomass is harvested by centrifugation and stored at a temperature of ≤ −60°C. A portion of the biomass is thawed, and the insoluble cell-bound Hyr1 is chemically and mechanically lysed from the cells through homogenization and centrifugation to isolate the inclusion bodies, which are then washed for impurity reduction before being subjected to tangential flow filtration for protein refolding. Once refolded, the Hyr1 is flowed through an anion exchange QFF resin and then captured and eluted from a cation exchange MegaCap II SP550 column. The eluate is then concentrated and diafiltered by TFF, flowed through a Mustang E anion exchange membrane for impurity reduction, 0.2μM filtered and frozen at ≤ −60C (Fig. 1).

### Adjuvants

We used CAF01, BDX100, and Alum adjuvant in this study. CAF01 is a two-component liposomal suspension composed of N, N’-dimethyl-N,N’-dioctadecylammonium bromide (DDA), and α’-trehalose-6,6’-dibehenate (TDB) and is being developed by Serum and Statens Institut, Denmark. CAF01 is prepared by forming thin lipid films containing DDA and TDB in a 5:1 (w/w) ratio, followed by hydration in a buffer solution, resulting in liquid crystalline bilayer vesicles. BDX100 (Protolin) consists of *Neisseria meningitidis* outer membrane protein (OMP) non-covalently associated with *Shigella flexneri* lipopolysaccharide (LPS) in a 1:1 ratio. BDX300 (V2 Proteosome) consists of *N. meninigitidis* Omp and LPS (Inspirevax, Kirkland, QC, Canada). Alum (Aluminum Hydroxide, Adjuphos) and MF59 (Novartis Proprietary adjuvant) were sourced from Inovio. MF59 is an Oil-in-Water emulsion (squalene, Tween 80, and Span 85 surfactants)-based adjuvant known to induce Th2 and humoral antibody response[96]. Glucan Chitosan Particles were prepared from fungus *Rhodotorula mucilaginosa* [63], loaded with Als3p or Hyr1p antigens, and administered separately subcutaneously.

### Vaccine Formulations

The final formulated (Als3 + Hyr1 + adjuvant) vaccine consists of two separately purified recombinant antigens and one of the adjuvants (Alum, CAF01, BDX100, BDX300, MF59, and GCP). We used a checkerboard method to obtain different antigen ratios by combining Als3p antigen at 0, 10, or 30 μg/dose with either 0, 3, 10, or 30 μg/dose of Hyr1p. Monovalent vaccine formulations with Als3 or Hyr1 alone were used to compare antibody and T cell development to dual antigen formulations and any potential immunodominance by one antigen over the other.

For each vaccine dose, different antigen ratios of Als3p and Hyr1p antigens were mixed with 200 μg of Alum (Inovio), 300 μg of a two-component liposomal adjuvant system (CAF01; Croda International Plc), 100 μl of MF59, 50 μg of BDX100, or 50 μg of BDX300 adjuvant. Als3p or Hyr1p antigens were encapsulated in 200 ug (1×10^8^ particles) Glucan Chitosan particles (GCP). The volume for each vaccine dose was adjusted to 0.2 ml with diluent Phosphate buffer saline (pH 7.4).

### Mice Vaccination

The ICR CD-1 4–6 weeks old mice were vaccinated with the formulated vaccine candidates on days 0 and 21 or days 0, 21, and 35 subcutaneously (SC) or intranasally (IN) (for BDX100 and BDX300 formulations only). The mice were euthanized two weeks after the final vaccination for immunogenicity determination, and sera and spleens were collected. Sera were used to evaluate anti-Als3 and Hyr1 IgG antibody endpoint titers by ELISA. Splenocytes were used for FluroSpot assay to determine the frequency of Als3 or Hyr1 antigen-specific Th1, Th2, or Th17 cells. The mice were infected two weeks after the final vaccination for infection experiments.

### Antibody Titer Determination

Polystyrene 96-well plates were coated with 5 μg/ml of Als3p or Hyr1p in 1X PBS buffer (pH 7.4) and incubated overnight at 4°C. The following day, the plates were washed three times with 1X wash buffer (PBS with 0.05% Tween-20) and blocked with 1% BSA solution for 2 hours at room temperature. After another three washes, diluted serum samples were added in duplicates and incubated for 2 hours. Post-incubation, the plates were washed three times, and 1:1000 diluted anti-mouse IgG antibodies (Jackson, Cat#115-035-164) labeled with peroxidase were added and incubated for 1 hour at room temperature. Finally, the plates were washed five times with wash buffer, TMB substrate (Invitrogen, Cat#00-4201-56) was added, and color development was allowed for 5–10 minutes. Absorbance was measured at 450 nm after stopping the reaction with 1 N sulfuric acid (Sigma, Cat#339741) [75].

### FluroSpot Assay

We employed a CTL^™^ IFN-γ/IL-4/IL-17 triple color FluoroSpot assay kit (CTL ImmunoSpot, Cleveland, OH) to assess antigen-specific T cell immune responses. The FluoroSpot assay plates were prepared by activating the membrane with ethanol and washing with PBS. Mouse Cytokine Capture Solution was prepared according to the manufacturer’s instructions and added to the plates. The plates were incubated overnight at 4°C and then washed with 1X PBS.

Spleens from vaccinated animals were collected and individually processed by homogenizing through a 100 μm cell strainer. RBCs were lysed using 1x RBC lysis buffer (Santa Cruz Biotech, Dallas, Cat# SC-296258) and filtered through 100 μm sterile filters. The cells were resuspended in CTL serum-free medium, counted, and plated at 3×10^5^ splenocytes/0.1 ml/well. The splenocytes from each mouse were either left unstimulated or stimulated with 0.1 ml/well Als3p or Hyr1p at 10 μg/ml, or with mitogen (10 ng/ml Phorbol myristate acetate [PMA]/250 ng/ml Ionomycin) along with anti-CD28 antibody.

The plates containing antigens and splenocytes were incubated for 24 hours at 37°C. After incubation, cytokine spots were developed using an anti-mouse IFN-γ/IL-4/IL-17 cytokine detection solution, followed by a tertiary solution. The developed FluoroSpots were air-dried, imaged, and counted using the CTL ImmunoSpot plate reader. FluoroSpots in the unstimulated wells of each mouse were subtracted from the antigen-stimulated spot counts and graphed.

### Infectious Inoculum Preparation

The *C. albicans* reference strain SC5314 (ATCC-MYA-2876) and *C. auris* strain CAU-09 (South Asian clade, bronchoalveolar lavage [BAL]) were used in this study. These strains were grown in Yeast Extract Peptone Dextrose (YPD) broth overnight at 30°C with shaking at 200 rpm. Yeast cells were washed with 1x phosphate-buffered saline (PBS, Gibco by Life Technologies) three times prior to counting blastopores with a hemocytometer. For intravenous injection, *C. albicans* and *C. auris* inoculum were adjusted to 5X10^7^ cells/0.2 ml and 2X10^5^ cells/0.2 ml, respectively. For vaginal infection, *C. albicans* inoculum was adjusted at 1×10^8^ cells/mL [66, 75, 97, 98].

### Mice Infection and Treatment

ICR CD-1 mice were intravenously infected two weeks after vaccination with either *C. albicans* or *C. auris*. For *C. albicans* infection, 2×10^5^ cells/0.2 ml were administered via tail vein injection. For *C. auris* infection, mice were immunosuppressed with 200 mg/kg cyclophosphamide (i.p.) and 250 mg/kg cortisone acetate (s.c.) given on day − 2 relative to infection. To prevent bacterial superinfection, enrofloxacin (50 μg/ml) was added to the drinking water and continued until day 7 post-infection. Mice were then intravenously injected with 5×10^7^ cells/0.2 ml of *C. auris* [75].

For the antifungal and vaccine combination treatment, vaccinated and infected mice received a minimal protective dose of 2.0 mg/kg/day of Fluconazole for *C. albicans* and 0.5 mg/kg/day of micafungin (i.p.). for *C. auris*. Treatment began 24 hours post-infection and continued until day + 7. Mice were monitored for survival over 21 days post-infection.

For vaginal infection, vaccinated mice received a 1.6 μg/gram mouse weight dose of β-Estradiol 17-valerate (Sigma, Cat# E1631–1G) before and during infection with *C. albicans*. β-Estradiol 17-valerate was administered subcutaneously at 0.1 ml/mouse in the back of the neck on days − 3, 0, and + 3 relative to infection [66].

To determine fungal burden, mice were euthanized on day 4 (day 5 for vaginal infection) post-infection to collect kidneys, hearts, and brains (vaginal tissues for vaginal infection). The organs were weighed, homogenized, and quantitatively cultured using 10-fold serial dilutions on YPD plates. Plates were incubated at 37°C for 48 hours before enumerating colony-forming units (CFUs)/gram of tissue [66, 75].

Histopathological examination of kidneys, hearts, and brains from mice sacrificed on day 4 post-infection involved fixing the tissues in 10% zinc-buffered formalin, embedding in paraffin, sectioning, and staining with Hematoxylin and Eosin [75].

### Antibody Adoptive Transfer and T Cell Depletion Studies

The mice were vaccinated as described above and grouped as depletion and control depletion arms. For CD4 + T cell depletion, 200 μg/mouse dose of rat anti-mouse CD4 IgG2b (clone GK1.5, BioXcell, Cat #BE0003–1)) or rat IgG2b isotype antibodies (Clone: LTF-2, BioXcell, Cat #BE0090) were administered intraperitoneally on day − 3 and 0 relative to infection. Mice were infected intravenously with either *C. albicans* or *C. auris* and monitored for their survival for three weeks.

Three additional mice were taken in each depletion and control depletion arm to verify the CD4 T Cell depletion 4 days after administering the second dose of the depletion drug. The mice were euthanized, and their spleen and inguinal lymph nodes were harvested and homogenized to make a single-cell suspension.

The sera were collected and pooled from the vaccinated animals for Antibody adoptive transfer. Naïve mice infected with *C. albicans* or *C. auris* as above and treated with either serum from vaccinated mice or placebo mice on days 0 and 7. The mice were monitored for survival [75].

### Flow Cytometry

Splenocytes were stained with anti-CD3 APC (BD Pharmigen, Cat #BDB565643) and anti-CD4 Alexa Fluor 700 antibodies (Biolegend, Cat #100536). The stained cells were acquired in a BD LSR II flow cytometer, and data were analyzed in FlowJo V10 software.

### Statistical Analysis

Survival differences were analyzed using the non-parametric Log Rank test for overall survival and Mantel-Cox comparisons for median survival times. All other comparisons were performed using the non-parametric Mann-Whitney test. A p-value of < 0.05 was considered significant.

## Supplementary Material

Supplementary Files

This is a list of supplementary files associated with this preprint. Click to download.

- SUPPLEMENTALDATAFILE07.22.25.docx
- Table1.docx

## Data Availability

The data are available in the main text or the supplementary materials of this manuscript.
